# Pluripotent Stem Cell-Derived In Vitro Gametogenesis and Synthetic Embryos—It Is Never Too Early for an Ethical Debate

**DOI:** 10.1093/stcltm/szad042

**Published:** 2023-07-20

**Authors:** Stefanie Horer, Michael Feichtinger, Margit Rosner, Markus Hengstschläger

**Affiliations:** Institute of Medical Genetics, Center of Pathobiochemistry and Genetics, Medical University of Vienna, Vienna, Austria; Wunschbaby Institut Feichtinger, Lainzerstraße 6, Vienna, Austria; Institute of Medical Genetics, Center of Pathobiochemistry and Genetics, Medical University of Vienna, Vienna, Austria; Institute of Medical Genetics, Center of Pathobiochemistry and Genetics, Medical University of Vienna, Vienna, Austria

**Keywords:** in vitro gametogenesis, synthetic embryos, in vitro fertilization, ethics, genome sequencing, artificial intelligence

## Abstract

Recently, 2 branches of the wide area of synthetic biology—in vitro gametogenesis and synthetic embryo development—have gained considerable attention. Rodent induced pluripotent stem cells derived via reprogramming of somatic cells can in vitro be differentiated into gametes to produce fertile offspring. And even synthetic embryos with organ progenitors were generated ex utero entirely from murine pluripotent stem cells. The use of these approaches in basic research, which is rightfully accompanied by an ethical discussion, will allow hitherto unattainable insights into the processes of the beginning of life. There is a broad international consensus that currently the application of these technologies in human-assisted reproduction must be considered to be unsafe and unethical. However, newspaper headlines also addressed the putatively resulting paradigm shift in human reproduction and thereby raised expectations in patients. Due to unsolved biological and technological obstacles, most scientists do not anticipate translation of any of these approaches into human reproductive medicine, if ever, for the next 10 years. Still, whereas the usage of synthetic embryos for reproductive purposes should be banned, in the context of in vitro-derived human gametes it is not too early to initiate the evaluation of the ethical implications, which could still remain assuming all technological hurdles can ever be cleared.

## Graphical Abstract

**Graphical Abstract:**
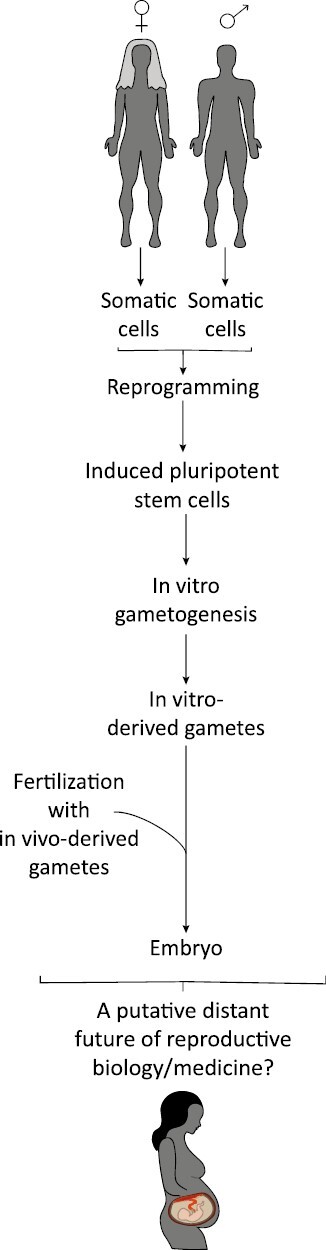

**Significance Statement**

A variety of major technological hurdles would have to be overcome before in vitro gametogenesis could ever find its way into human reproductive medicine. This perspective discusses the recent scientific breakthroughs in this field and points out the importance to initiate an international ethical discussion about the usage of human in vitro gametogenesis without any delay.

## Introduction

Since in vitro fertilization (IVF), a technological innovation recognized by the Nobel Prize in Physiology and Medicine to Robert Edwards in 2010, came to the fore in 1978 it sparked a remarkable development in human reproduction. The latest world report on assisted reproductive technology comes to the conclusion that in the year 2018 alone about 770 000 babies were born resulting from about 3.2 million IVF approaches in 79 countries.^1^ This development was driven by both the permanent improvement of assisted reproduction technologies and a significant increase in the prevalence of infertility in the Western world. More than every 10th person of reproductive age was willing to conceive is affected by infertility, a condition defined upon verification of the failure to achieve a pregnancy after a period of 1 year of unprotected sexual intercourse or of a specific impairment of a patient’s capacity to reproduce.^2-4^ The reasons for finding oneself in the situation of an unfulfilled desire to have children are manifold. A significant proportion is characterized by the absence of available gametes. Premature ovarian failure, ovarian cancer and gonadotoxic anti-cancer therapies, polycystic ovary syndrome, and age-related factors such as a declined ovarian reserve in the context of an increasing trend to advanced maternal age reproduction can cause the lack of functional eggs. In men, the absence of sperm in the ejaculate is caused by cytotoxic cancer therapies or much more frequently by nonobstructive azoospermia as a consequence of genetic, inflammatory, or endocrine disorders.^4^Infertile men and women as well as gay couples commonly resort to the usage of donated sperm or eggs for assisted reproduction or to adoption. However, so far the entire spectrum of assisted reproduction technologies cannot help such advice-seeking individuals to conceive genetically related children.^2-4^

In the last months, in media reports, it was circulated that pluripotent stem cells (PSCs) could be a magic bullet to solve this problem. Although both PSC-derived in vitro gametogenesis (IVG) and synthetic embryos are a long way from any kind of human medical application recent groundbreaking scientific reports triggered media outlets with headlines which initiated a wave of enthusiasm accompanied by a pronounced public discussion of ethical concerns.^5-10^There is no doubt that these innovative technological approaches offer potential benefits in basic research and in the clinical context. As scientists studying human PSCs^11^ we are fascinated to see the opportunities that open up for basic research especially since many congenital and late-onset diseases have their roots in deregulated early embryogenesis.^12^ As clinicians working in the IVF field also performing preimplantation genetic diagnosis (PGD)^13^ we are constantly confronted with the wishes and expectations of patients yearning for their genetic offspring. Accordingly, we want to highlight that for the benefit of patients these scientific findings should—as far as possible—realistically be assessed to avoid overinterpretation and to allow an ­evidence-based discussion of the question “What might come next and when?.” And especially in the context of the ethical ­evaluation of IVG, we should already start to debate the pros and cons in order to be prepared for the time when the technological obstacles regarding efficacy and safety are removed.

## IVG on the Route to Translation

PSC-derived IVG was originally established in mice. Murine PSCs have been differentiated into primordial germ cell-like cells (PGCLCs), were further developed into eggs and sperm both upon introduction into ovaries or testes and in specific cellular environments, and have finally been used to generate viable offspring.^14-17^ Based on the success of years of research on mouse PSC-derived gametes it was recently shown that functional PGCLCs capable of siring viable rat offspring can also be derived from rat PSC.^18^ These data argue for the broad applicability of IVG in mammals. At least from the point of view of the media all that was surpassed by the recent finding that male somatic cells can be coaxed to become eggs. Somatic cells from an adult male mouse were reprogrammed into induced PSCs (iPSCs), grown until they spontaneously lost their Y chromosomes, and treated with the compound reversine known to promote chromosome errors to generate iPSCs with 2X chromosomes. Subsequently, these iPSCs were driven to produce eggs, which were fertilized with mouse sperm and transferred into the uterus of a female mouse leading to the birth of mice with 2 dads.^19^

**Figure 1.**
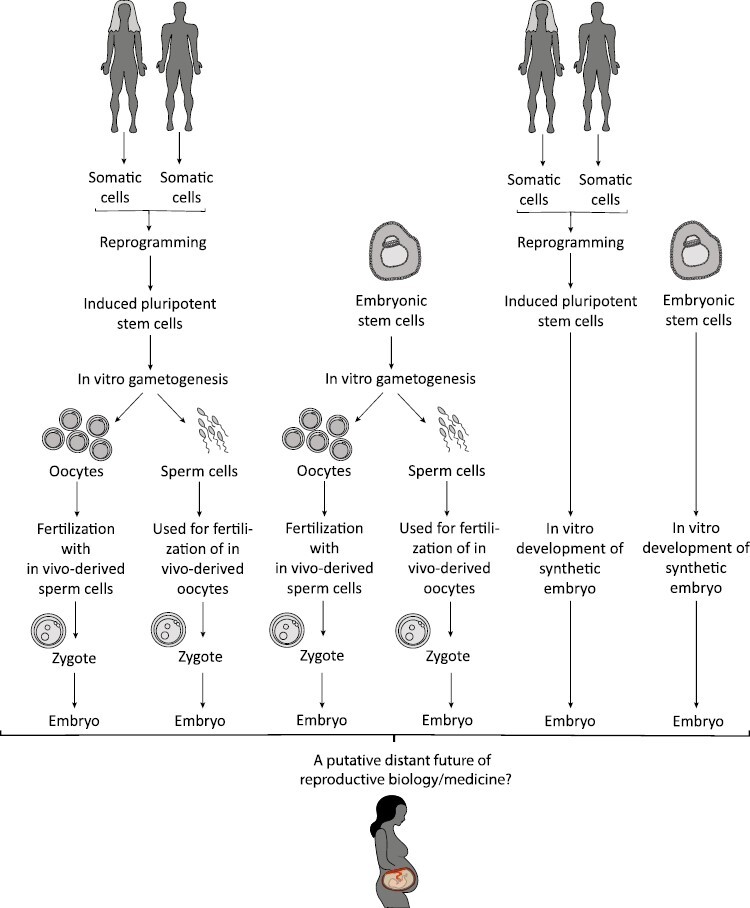
Schematic representation of selected hypothetical strategies using in vitro gametogenesis and synthetic embryos in the distant future. The designation “in vivo-derived” is used for natural existing gametes. For many reasons, the usage of synthetic embryos for assisted reproduction is considered to be unethical. And also in vitro gametogenesis for human reproductive purposes is currently categorized as a prohibited research activity until safety and ethical issues are resolved (for details see the text).

Since it has also already been shown that rudimentary human oocytes^20^ and sperm^21^ can be generated from human PSCs by cultivation with mouse ovarian or testicular cells, the question concerning the first occurrence of a human pregnancy upon IVG is not too far-fetched (Fig. 1). Whereas the International Society for Stem Cell Research (ISCCR) has categorized IVG for human reproductive purposes as a currently prohibited research activity until safety and ethical issues are resolved (category 3A)^22^ it has also already been speculated that one day a large proportion of human pregnancies will result from IVG.^23^ In this context it is noteworthy that a variety of companies, which work on the translation of human IVG to the clinic, have already obtained remarkable venture capital funding.^24^ The still existing biological hurdles prompted most scientists to not anticipate the application of human IVG in assisted reproduction procedures for at least a decade.^5,24^ The major concerns are associated with the currently unforeseeable but in future probably determinable genetic and epigenetic aberrations in in vitro-derived gametes and IVG-derived embryos. Generally speaking, the genetic and epigenetic integrities of in vitro-derived gametes and germ cells appear to be substantially lower compared to their in vivo counterparts.^25^ Such aberrations can be acquired in the course of the IVG process by failures regarding imprint erasure, imprint resetting, or meiosis. Furthermore, they can also be inherited from the somatic parent cells knowing that the mutation rate in somatic cells is 10-fold higher than in germ cells.^26^ Indeed, mouse PSC-derived gametes and germ cells have been demonstrated to be of lower quality than their in vivo counterparts.^16,27^ In line with this the survival rate of IVG-derived mouse embryos has been observed to be low so that. for example, only 7 out of 630 transferred embryos survived in the course of the recent study generating functional oocytes from male mice.^19^ Many IVG-derived mouse embryos die prenatally and the surviving animals have been discussed to harbor cryptic anomalies.^16,25^ Accordingly, the current ISSCR categorization of IVG is well justifiable considering this spectrum of unsolved technological problems and unanswered biological questions but also suggests that the time of human IVG in human reproduction can come once these obstacles have been removed.^22^ In general, the first strategies that are considered to be particularly promising to pave the way for IVG to human reproduction include the usage of reserved fetal/neonatal cells harboring fewer mutations together with genetic and epigenetic assessments of the human PSCs as well as of the generated gametes^25^ and, for example, for same-sex reproduction an alternative approach using ESC-derived gametes of the opposite sex has also been suggested.^28^

## Synthetic Embryos and Human Reproduction—An Unimaginable Vision

International media hype was also provoked by the recent demonstration that synthetic mouse embryos developing organ progenitors such as brain and heart together with complex extra-embryonic compartments could be developed ex utero entirely from murine PSCs.^7-10,29,30^These groundbreaking technologies will allow new important insights to obtain a more comprehensive picture of early mammalian embryogenesis. Failures of embryo implantation or aberrations of embryonic development have been estimated to occur in up to 50% of human pregnancies and many human diseases have their roots in deregulations concerning the embryonic period of development.^12,31^ However, due to the scarcity of human tissue material and the inaccessibility of the in vivo condition the knowledge about early human embryogenesis is still extremely limited. In addition, in many countries laws exist which prohibit or severely restrict research on human embryos. Such legislations often result from the local prevailing opinion about the moral status of the human embryo and its protection status.^32-36^ Furthermore, knowledge derived from murine synthetic embryos cannot necessarily directly be assigned to humans, because of the well-described significant interspecies differences regarding embryogenesis.^37^ Accordingly, independent of the obviously indispensable ethical discussion which should accompany this research, the question of whether the same technological approaches could potentially be used to generate PSC-derived human synthetic embryos must currently remain elusive. Due to the difference in complexity between mouse and human embryos, it has been argued that it could be decades before human synthetic embryos could successfully be generated.^10^

Since these murine synthetic embryos have been derived from PSC via bypassing the need for sperm, eggs, and fertilization,^29,30^ the discussion about the usage of these technologies in support of human reproduction in future could theoretically be initiated.^22^ And again, from a biological point of view genetic and epigenetic aberrations (either inherited from the parent cells or introduced in the course of the generation process), could lead to implantation failure, pregnancy loss, or the birth of children with developmental disabilities, would move into focus. In addition, the existing approaches have led to the generation of synthetic mouse embryos, which clearly show differences, deviations, and aberrations when stringently compared to natural embryos.^29,30^ Accordingly, there is currently no foreseeable getting around to categorizing such attempts as prohibited research activities (ISCCR, category 3B): The transfer of human stem cell-based embryo models to the uterus of either a human or animal host “should not be pursued because of broad international consensus that such experiments lack a compelling scientific rationale and are widely considered to be unethical.”^22^ In this context it is very important to note that current studies aim to use human embryo models exclusively for research applications and that this research is seen to be associated with less ethical concerns because these embryo models do not have the potential to develop into human beings.^32,35,36^

## Conclusion: The Urgent Need for an Ethical Debate

From today’s perspective, it is widely argued that PSC-derived synthetic embryos should not be used in human reproductive medicine, even not in the unlikely event that all safety issues could ever be resolved.^22^ In the context of other assisted reproduction technologies some degree of remaining risk seems obviously to be acceptable and the focus is rather on the question “How safe is safe enough?.” With regard to PSC-derived synthetic embryos, one could as well emphasize the existing coincidences with the ethical discussion regarding human reproductive cloning, also classified as research category 3B, and about the moral status of the embryo.^12,22,33-36,38^Already in 2018, a comprehensive ethical comparison of IVG and reproductive cloning has been published, including the discussion to which extent the advancement of one technology affects the ethical evaluation of the other.^39^ However, finally it is important to highlight that the ban on the usage of PSC-based entities for reproductive purposes has already been demanded years before the publication of the recent reports on synthetic embryos.^32^

In contrast, IVG could be used in future to treat infertility in the course of assisted reproduction as soon as it has been proven to be safe. Although a specific date for the first application of human IVG cannot be foreseen it probably won´t happen much later than in 10 years.^24,25^ Accordingly, we feel an international debate about the social, ethical, and legal consequences, which should build the basis for the establishment of well-applicable legal framework conditions, to be overdue.

First, an agreed-upon general naming convention should be put in place. In agreement with many scientists, we want to recommend the usage of “in vitro-derived gametes” rather than “artificial gametes” or “synthetic gametes,” because it is less value-laden and less negatively connoted. Also from our personal experience, we would expect that terms such as “artificial” or “synthetic” could definitively hamper the patients´ acceptance of such approaches.^40,41^ The avoidance of “artificial” or “synthetic” for PSC-derived embryo models has also been suggested.^22^ However, one group of authors, who reported the successful generation of these embryo models, use the term “synthetic embryo” themselves,^29^ which prompted us to use it in this article. Although in a broader sense both in vitro-derived gametes and synthetic embryos could probably be subsumed under “synthetic biology,” one could argue that these two entities differ with regard to the process of generation. Whereas the generation of in vitro-derived gametes mimics the in vivo process including differentiation and meiosis,^25,42^ the genesis of embryos developed without eggs, sperm, and fertilization^29,30^ does not have a natural counterpart. This could shed another light on the usage of the term “synthetic.”

Hypothetically, a large variety of applications for IVG exist and their moral advantages and disadvantages together with, for example, social consequences must be discussed. In Fig. 1 some putative strategies are depicted. For traceable reasons, IVG originating from iPSCs rather than from embryonic stem cells (ESCs) is predominantly discussed in the context of the translation to the clinic, especially with regard to infertility treatment. Whereas it could also be considered to use in vitro-derived gametes from both the mother and the father if both parents lack functional gametes, the mixed usage of in vitro-derived and natural existing in vivo-derived gametes (as depicted in Fig. 1) might more likely come into effect in the daily clinical work. In addition to these opposite-sex applications to treat infertility, the recent report on the generation of oocytes derived from male somatic cells, forms the basis for speculations about so-called same-sex reproduction via IVG with the aid of surrogate mothers.^5^ Beside same-sex reproduction, the possibility to create complementary gametes to those an individual already has triggered the discussion about prospective electively single parents. Irrespective of whether and under which experimental conditions this could ever be implemented in reality, such approaches have already found their way into the literature on ethics under the designation “solo reproduction” since they would raise serious biological and ethical concerns.^5,43,44^

In general, many arguments in favor of and against IVG in human reproduction can be collected and weighed. We would like to refer to already published in-depth contemplations on this topic.^28,39-41,43,44^ Without a claim of completeness and just by way of illustration we want to raise some aspects: The involuntary inability to conceive genetically related children can take a toll on a patient’s well-being, for example, by lowered self-esteem. Putative individual ethical doubts associated with adoption or gamete donation could be eluded. The burden of stimulation and invasive oocyte puncture could be avoided. And it could be a reproductive chance for women at an advanced age. Otherwise, this could also result in pressure on women to give birth at an advanced age. In addition, the costs of IVG in the course of assisted reproduction could turn it into a luxury item as part of undesirable 2-class medical systems.^28,39-41,43,44^

The next years of human IVG research will be driven by efforts to surmount the major biological obstacles which could finally culminate in both solving relevant issues and raising new concerns. Although the employment of different technologies for the genomic assessment of embryos is still in its infancy this field of research is very innovative and growing rapidly.^45,46^ For a putative prospective usage of IVG in human reproductive medicine one could assume that several IVG-derived embryos would have to be screened to identify candidates for the transfer into the uterus. Today, the hormone stimulation regimen to mature a sufficient number of capable oocytes for IVF is of the highest impact on its success rate. In future, the appeal of high numbers of eggs derived from iPSCs without ovary stimulation could be that numerous embryos could genetically be screened. On the one hand, this could be the door opener for the application of human IVG. On the other hand, having the option to choose between so many embryos might reflect a paradigm shift that could overstrain patients. The recent developments in preimplantation genetic testing for polygenic risk (PGT-P) have already initiated an extensive debate regarding the technological challenges, significance, and ethical consequences of screening embryos for polygenic conditions and traits.^47-49^ In general, it is the hope of patients and clinicians alike that the convergence of artificial intelligence (AI) and genome sequencing technologies will allow for a much more comprehensive picture of polygenic diseases triggering the development of many innovative therapeutic strategies.^50^ However, with the prospect of a putatively successful clinical implementation of human IVG this convergence could be assumed to give many more answers but also raise many more questions at the same time (for detailed discussion see references ^28,39-41,43,44,47-49^). The initiation of an international ethical debate regarding the screening of many human embryos as a perspective of putative prospective employment of IVG in human reproductive medicine appears also to be of importance considering the results of a recent nationally representative US survey-based experiment on the attitudes towards PGT-P: Over 50% of the participants did not see a moral issue or felt it to be morally acceptable to use this technology to increase the chance of having a child which attends a top-100 college by 2% points.^51^ However, last but not least, it is important to note that this ethical debate has to take into consideration that IVG could once help advice-seeking individuals to conceive healthy genetically related children.

## Data Availability

No new data were created or analyzed in support of this study.
